# The Bunyavirales: The Plant-Infecting Counterparts

**DOI:** 10.3390/v13050842

**Published:** 2021-05-06

**Authors:** Richard Kormelink, Jeanmarie Verchot, Xiaorong Tao, Cecile Desbiez

**Affiliations:** 1 Laboratory of Virology, Department of Plant Sciences, Wageningen University, Droevendaalsesteeg 1, 6708 PB Wageningen, The Netherlands; 2 Department of Plant Pathology and Microbiology, Texas A&M University, College Station, TX 77843, USA; jeanmarie.verchot@exchange.tamu.edu; 3 Department of Plant Pathology, Nanjing Agricultural University, Nanjing 210095, China; taoxiaorong@njau.edu.cn; 4 INRAE, Pathologie Végétale, F-84140 Montfavet, France; cecile.desbiez@inrae.fr

**Keywords:** Tospoviridae, Orthotospovirus, Phenuiviridae, Tenuivirus, Fimoviridae, Emaravirus, Tomato spotted wilt virus, TSWV, Rice stripe virus, RSV, European mountain ash ringspot-associated virus, EMARaV

## Abstract

Negative-strand (-) RNA viruses (NSVs) comprise a large and diverse group of viruses that are generally divided in those with non-segmented and those with segmented genomes. Whereas most NSVs infect animals and humans, the smaller group of the plant-infecting counterparts is expanding, with many causing devastating diseases worldwide, affecting a large number of major bulk and high-value food crops. In 2018, the taxonomy of segmented NSVs faced a major reorganization with the establishment of the order *Bunyavirales*. This article overviews the major plant viruses that are part of the order, i.e., orthospoviruses (*Tospoviridae*), tenuiviruses (*Phenuiviridae*), and emaraviruses (*Fimoviridae*), and provides updates on the more recent ongoing research. Features shared with the animal-infecting counterparts are mentioned, however, special attention is given to their adaptation to plant hosts and vector transmission, including intra/intercellular trafficking and viral counter defense to antiviral RNAi.

## 1. Introduction

**Figure 1:**
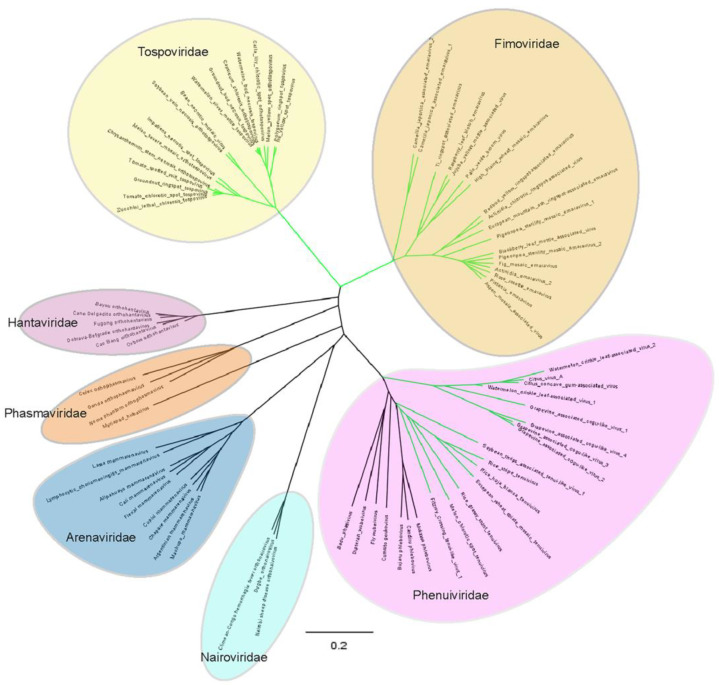
Phylogeny of seven major families within Bunyavirales in terms of the RdRp. Displayed is an unrooted neighbor joining (NJ) tree. Three families that include plant-infecting viruses are identified by green branches. The plant viruses of *Tospoviridae* and *Fimoviridae* share closer relationship than plant-infecting members of *Phenuiviridae.* A more comprehensive analysis of the family-level phylogenies within Bunyavirales is provided in Herath et al. [23].

Negative-strand RNA viruses (NSVs) have historically been classified into two major groups, those with non-segmented genomes (order *Mononegavirales*) and those with segmented genomes. Plant viruses, although being a relative minority of NSVs, have representatives in both groups within the families *Rhabdoviridae* and *Bunyaviridae*, and several floating genera (*Tenuivirus*, *Ophiovirus*, *Emaravirus*, and *Varicosavirus*). The first overview of the plant-infecting viruses among the NSVs was published a decade ago [1], but research has progressed, new viruses have been discovered, floating genera have been upgraded to the family level, and reverse genetics systems have been developed. Furthermore, in 2018, the taxonomy of NSVs faced a major overhaul and reorganization based on observed homology between the viral RNA-dependent RNA polymerases (RdRp) with, amongst others (Table 1), the establishment of the new order *Bunyavirales*, including the families *Bunyaviridae* and *Arenaviridae* and several newly established families. Within the order there are three newly established plant virus families: *Fimoviridae* (an upgrade of the *Emaravirus* genus), *Phenuiviridae* (siglum/merge and upgrade of the former *Phlebovirus* and floating *Tenuivirus* genus into one family), and *Tospoviridae* (an upgrade of the former genus *Tospovirus*, now *Orthotospovirus*, previously part of the former *Bunyaviridae*). The establishment of these families within the order, and the merging of tenuiviruses and phleboviruses, is supported by phylogenetic analyses (Figure 1). In addition to that, two new genera have very recently been established within the *Phenuiviridae*, comprising new and distinct NSV plant viruses with segmented genomes, i.e., *Coguvirus* and *Rubodvirus* (Table 1).

With this taxonomy update, latin binomials have also been introduced. However, scientists have preferred species to be recognized as the primary subjects of virology, in analogy to common practices elsewhere in biology [2].

In the following sections, a brief history and introduction to the families *Tospoviridae*, *Phenuiviridae*, and *Fimoviridae* will be given, followed by a detailed description and update on their molecular biology and replication cycle, as well as the role of non-structural accessory proteins in host adaptation, transmission, and evolution. The overview will be closed with some perspectives and challenges faced for the plant-infecting members of the Bunyavirales.

### 1.1. Tospoviridae: Genus Orthotospovirus

It was not until the 1990s of the past century when *Tomato spotted wilt virus* (TSWV) became recognized as the first plant-infecting virus of the *Bunyaviridae*, within the genus *Tospovirus* (siglum from tomato spotted) [3]. At first, it was the sole species of the genus, but with the development of molecular tools and diagnostics, the genus soon expanded and by now has grown to almost 30 established and tentative species. Nowadays tospoviruses are widely distributed and found in many agricultural, horticultural, and ornamental crops (dicots and monocots) [4]. With the establishment of the order *Bunyavirales*, the genus *Tospovirus* has been fully upgraded to the family *Tospoviridae*, which currently holds one genus, *Orthotospovirus*.

Species demarcation relies on amino acid (aa) sequence identity (<90%) of one of its major structural proteins, i.e., the nucleo(capsid) protein (N), with all other established orthotospoviruses. Whereas this is the major demarcation criterium and led to the recognition of the two major American and Euro-Asian clades of orthotospoviruses, the species are often biologically distinguished additionally by their host range and vector specificity. Orthotospoviruses are transmitted in a persistent manner, in which the virus also replicates in the insect vector thrips (Family *Thripidae*). Approximately 15 different thrips vectors have been identified to transmit orthotospoviruses, although some have a limited geographical distribution [4,5,6].

Although tomato spotted wilt disease was already reported more than 100 years ago in Australia, it was not until the 1980s that TSWV rapidly spread due to the worldwide expansion of *Frankliniella occidentalis*, the Western flower thrips, one of its major thrips vectors [5,7]. Subsequently TSWV has become one of the most important and devastating plant viruses worldwide [8], and many review papers have already appeared on orthotospoviruses, often with special emphasis on certain issues such as the molecular biology, host defense mechanisms, epidemiology, and vector transmission,. Readers are also referred to other articles for additional reading [4,5,6,9,10,11,12]. Although the sections further below will generally speak on orthotospoviruses, most information is taken from studies on TSWV, unless stated otherwise.

### 1.2. Family Phenuiviridae: Genus Tenuivirus

Tenuiviruses constitute the major plant-infecting members of the family *Phenuiviridae*. Tenuiviruses have only been recognized as a plant virus group in the early 1980s, and at the beginning of 1990s were still reported to possibly contain a negative RNA genome. At that time, the group comprised five viruses: *Rice stripe virus* (RSV), *Maize stripe virus* (MStV), *Rice hoja blanca virus* (RHBV), *European wheat striate mosaic virus* (EWSMV), and *Rice grassy stunt virus* (RSGV). However, diseases caused by these viruses were already discovered and described as early as 1890 (RSV), 1929 (MStV), and 1935 (RHBV) [13].

Nowadays, the genus *Tenuivirus* comprises eight species, most of them infecting monocotyledonous hosts in the family *Poaceae*. RSV is the type species in the genus. Partial or complete sequence data suggest that other viruses, notably *Ramu stunt virus* (RmSV) affecting sugarcane in Papua New Guinea, and *Maize yellow stripe virus* detected on maize in Egypt, are distinct tenuivirus species, and metagenomic analyses suggest that a tenui-like virus may be present in the gymnosperm black spruce [14]. Tenuiviruses are transmitted by delphacid planthoppers in a circulative-propagative manner [15]. They generally induce the formation of white or yellow “stripes” in the leaves of infected plants, and they can severely reduce crop yield [16]. Tenuivirus-like disease symptoms have been observed in cereal crops for more than a century, whereas, in some of these cases, the role of a tenuivirus as the disease agent has only recently been confirmed [13,15,17].

RSV and RGSV constitute important problems on rice in Asia; RHBV, *Echinochloa hoja blanca* (EHBV) and *Urochloa hoja blanca virus* (UHBV) are present in the Americas only; MStV is the most prevalent, affecting mostly maize, in Africa, Asia, Central America, and Australia. *Iranian wheat stripe virus* has only been described in Iran. Melon tenuivirus (MeT) and *European wheat striate mosaic virus* (EWSMV, a tentative species) are present in Europe and have a minor agronomic impact. MeT differs from the other tenuiviruses because of its atypical genomic organization, efficient mechanical transmission [14], and being the first tenuivirus to infect dicotyledonous hosts.

### 1.3. Fimoviridae: Genus Emaravirus

Since the 1960s there have been reports of a ringspot disease in European mountain ash in several European countries, but it was not until the 1990s that the putative causal agent, a virus, was visualized using electron microscopy [18]. The virus, named European mountain ash ringspot-associated virus (EMARaV), provided the name to the genus *Emaravirus*, a taxon consisting of plant-infecting viruses. Emaraviruses have 5 to 10 genome segments. The pleiomorphic, double membrane-bound particles have a diameter of 80–200 nm enclosing all RNA segments that are bound by the N protein [19]. The most devastating viruses to natural environments, landscapes, and agriculture include *Pigeon pea sterility mosaic virus 1* (PPSMV-1), *European mountain ash ringspot associated virus* (EMARaV), *Rose Rosette virus* (RRV), and *High Plains wheat mosaic virus* (HPWMV) [20,21,22]. According to the ICTV, the genus comprises of 11 species, and peer reviewed literature searches identifies at least 18 more tentative species. The majority of the species identified infect Rosid and Asterid Eudicots, i.e., deciduous trees and shrubs [23]. Until now, only two species are known to infect monocots: HPWMV and *Ti-ringspot associated virus* (TiRsAV) [24,25]. Continuing high throughput sequencing (HTS) efforts by researchers globally has led to additional peer review reports of additional tentative species. These recent and expanding discoveries of new virus species that are likely members of the genus *Emaravirus* makes this an emerging genus whose global impact is not yet realized at the ecological or agronomic level.

## 2. Virion Composition, Genome Organization, and Intracellular Replication Cycle

### 2.1. Orthotospoviruses

**Figure 2:**
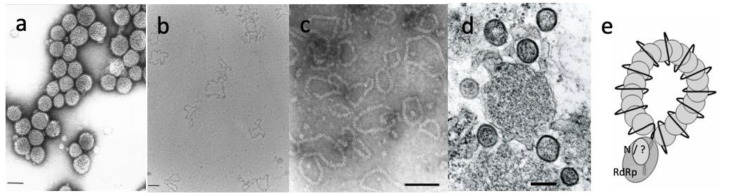
Transmission electron microscopy of TSWV particles ((**a**) courtesy of J. van Lent), TSWV ribonucleoproteins (RNPs) (**b**), RHBV tenuivirus particles (**c**), Emaravirus particles ((**d**) courtesy of K.S. Kim), and a schematical presentation of a viral ribonucleoprotein (RNP) (**e**). RdRp, RNA-dependent RNA polymerase (for tospoviruses also called L protein); N, nucleo(capsid)protein; potential other host-factors. Panhandle formed by complementary termini, and acting as promoter for replication–transcription, is shown in light gray at the position of the indicated N and RdRp. Size bar represents 100 nm (modified from [1]).

Orthotospoviruses consist of spherical, membrane-bound virus particles of approximately 80–120 nm in diameter (Figure 2a). The lipid envelope contains two viral envelope glycoproteins, Gn and Gc, that are produced by proteolytic processing from a glycoprotein (GP) precursor (n and c referring to the amino and carboxyterminal position within the precursor) and are instrumental in the acquisition and transmission by thrips vectors. The core of the virus particle contains the viral ribonucleo(capsid) proteins (RNPs) that consist of the viral RNA genome tightly enwrapped by nucleo(capsid) protein (N) and a few molecules of the viral RNA-dependent RNA polymerase (RdRp, also refered to as L protein) (Figure 2b,e).

**Figure 3:**
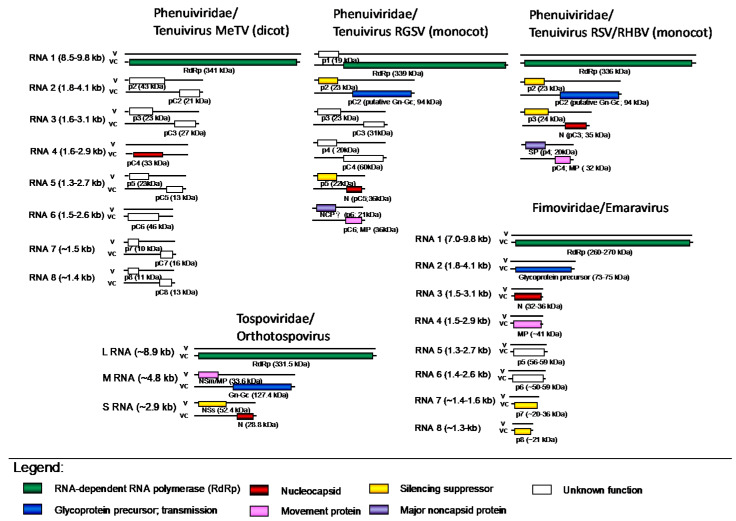
Genome organization of the plant-infecting representatives from the *Phenuiviridae* (genus *Tenuivirus*), *Tospoviridae* (genus *Orthotospovirus*), and *Fimoviridae* (genus *Emaravirus*) of the order *Bunyavirales*. Although emaravirus genomes contain 5 to 10 segments, as a reference genome HPWMV is shown. Functional homologous genes are indicated by color and indicated in the legend. v: viral RNA, vc: viral complementary RNA. Open reading frames (ORFs) are indicated by boxes. ORFs from ambisense RNA segments are expressed from sub-genomic length mRNAs, while ORFs from RNA segments of entire negative polarity are expressed from (near) genomic length mRNAs (modified from [1]).

The genome consists of three linear, single stranded (ss)RNA segments that, according to their sizes, are denoted Large (L, ≈9 kb), Medium (M, ≈4.8 kb), and Small (S, ≈2.9 kb) RNA (Figure 3). The L RNA is of entire negative polarity and encodes the RdRp (≈330 kDa) on the viral complementary (vc) strand (vc) [26]. Both M and S RNA contain an ambisense gene arrangement, i.e., encoding two non-overlapping reading frames (ORFs) on opposite strands that are separated by a non-coding intergenic region (IR) [27,28]. The M RNA encodes the non-structural cell-to-cell movement protein (NSm, ≈33 kDa) on the viral (v) strand and the GP precursor (≈128 kDa) on the vc strand [28,29]. Likewise, the S RNA encodes a second non-structural NSs protein (≈52 kDa) on the v-strand and the N protein on the vc strand [27] (Figure 3)

The 3′ ends of both genomic v and vc-RNA strands are highly conserved with nine nucleotides (3′-UCUCGUUA -5′) that are shared among all genomic elements of all orthotospoviruses. The 5′ end contains a highly conserved sequence that is inverted and complementary to the 3′ end, forming of a dsRNA panhandle structure and giving orthotospoviral genomic RNPs a pseudo-circular appearance (Figure 2b,e) [3,30]. The highly conserved sequences at the termini act as promoters for replication and transcription and extends with a segment specific sequence up to 15 nts. All proteins encoded by the RNA genome are expressed from near-genome length (L) or subgenomic length mRNAs (NSm, GP, NSs, N) transcribed from the genomic RNA [31]. Production of viral mRNAs involves a process of cap-snatching during which mature cellular host mRNAs are cleaved by the viral transcriptase complex about 12–18 nucleotides (nt) downstream the 5′-cap and used to prime transcription on the viral RNA template. Transcription termination of the subgenomic length M/S RNA-derived mRNAs occurs in the IR. This region is highly rich in A- and U-tracks and is predicted to fold into a stable hairpin structure that is postulated to act as transcriptional terminator [32]. As a result, viral mRNAs distinguish from viral (anti)genomic RNA molecules not only by the presence of a heterogeneous, non-viral sequence a the 5′ end but also by their size [33]. Other studies have indicated an additional role of the IR/hairpin structure in translational enhancement of viral mRNAs, in concert with N and NSs [34]. Orthotospoviral mRNAs lack a canonical poly(A)-tail, but also the 3′ end conserved genome segment terminal sequence. Furthermore, in contrast to (anti)genomic RNA molecules, viral mRNAs do not become encapsidated by the N protein, nor are they found within virus particles [31]. Crystal structure analysis, folding predictions, and RNA-N protein binding studies have shown that the N protein oligomerizes into an asymmetric trimeric ring in which the N and C-terminal (globular) arms of monomeric N mediate interaction to neighboring subunits. Genomic RNA is embedded within an inner cleft of the ring, which presents an RNA binding site, and in this way protects against cellular nucleases [35,36,37,38].

During the cytoplasmic replication of viral RNA, v-strands of the L RNA were observed in accumulating amounts during the course of infection, while the vc-strand was only observed in steady-state low levels. The ambisense M and S RNA v- and vc-strands were synthesized more equimolar, although the v RNA strands were produced in the highest amounts. Both strands of the M and S RNA were found in virus particles, although v-strands were observed most, whereas for the L RNA, only v-strands were found. Ratios of encapsidation in virus particles thereby seemed to reflect the amounts produced during cytoplasmic replication and support a random encapsidation mechanism [31,39].

Transcription–replication of the orthotospoviral RNA genome occurs in the cytoplasm and requires the RdRp and N proteins. The viral RdRp contains the conserved motifs and features of RdRp proteins typical from segmented NSV, i.e., an N-terminal endonuclease domain required for cap-snatching and a central domain containing the six motifs of RNA polymerase, including the typical “SDD” core motif. However, a cap-binding domain has not been identified yet and on this point also no structural sequence homology was observed with the cap-binding domain within PB2 of the influenza virus RdRp (composed of the three subunits PA, PB1, and PB2), nor within any other bunya/arenaviral/tenuiviral RdRp protein [40,41]. More recent 3D structural folding analysis and biochemical studies have shown the presence of a (putative) Cap-binding domain within the C-terminus of the RdRp of La Crosse peribunyavirus, Rift valley fever phenuivirus, and California Academy of Science reptarenavirus [42,43,44]. In case of the La Crosse RdRp, similar as within the influenza PB2 subunit, a protruding C-terminal domain was observed with the ability for large movements during transcription initiation [42,45,46].

As for the nuclear replicating orthomyxoviruses/influenza viruses, nascent mRNAs produced by the RNA polymerase II machinery present the source for capped-RNA leader sequences to initiate genome transcription [47,48], and the cytoplasmic replicating orthotospoviruses may likely use cytoplasmic RNA processing bodies (PB). *Arabidopsis thaliana* mutants, depleted from elements of the RNA decay machinery or nonsense-mediated decay pathway, exhibited increased susceptibility to TSWV infection [49]. Furthermore, TSWV N protein was observed to partially co-localize to P bodies. These observations match earlier observations made with Sin Nombre (SNV) hantavirus that showed first links to PB as a source of capped RNA for cap-snatching [50,51].

Viral replication is considered to occur at electron dense matter in the cytoplasm, in areas often designated as viroplasm [52], but whether and how these relate to the sites where transcription/cap-snatching occurs (P bodies?) is not known. After replication, progeny viral RNPs either move intra/intercellularly or mature into enveloped virus particles. To this end, RNPs receive their envelop membrane at the Golgi complex, as observed with most other animal-infecting viruses from the Bunyavirales, a process that involves multimeric protein interactions between the three major structural proteins (N, Gn, and Gc) and takes place at the ER and Golgi [53,54,55,56,57,58,59]. Viral Gn and Gc are produced by proteolytic processing of the GP precursor at the ER, most likely by ER-resident proteases, from where they concentrate at ER-export sites (ERES). Gc is only able to reach ERES by condensation with the cytosolic N protein [55]. Both mature envelope glycoproteins escape from ERES by COPII vesicle transport, requiring heterodimerization of Gc and Gn. The Gn glycoprotein contains a Golgi localization signal within its transmembrane domain (TMD) that allows for the escape and movement towards the Golgi complex by its own [54,56]. Intracellular movement of the mature envelope glycoproteins and N relies on actin filaments [54,60] and not on microtubules, as observed with the animal-infecting bunyaviruses in animal cells. Particle maturation occurs via wrapping of RNPs by an entire Golgi stack, in contrast to budding into the lumen of Golgi cisternae, as seen with most animal-infecting bunyaviruses [61,62]. Doubly enveloped virus particles resulting from this next fuse with each other and lead to the accumulation of mature (singly enveloped) virus particles in large vesicles. From there, the virus awaits uptake/acquisition by thrips upon feeding on the infected plants, and further disseminate [61].

### 2.2. Tenuiviruses

Viral particles are composed of non-enveloped ribonucleoproteins (RNPs), comprising ssRNAs encapsidated by the N protein. N monomers are able to self-interact, forming oligomers independently of RNA binding [63]. The predicted polar amino acids in the deep hydrophobic binding groove of the N protein are critical for RNA binding and to protect the RNA against RNAse digestion [63]. The RNPs are 3 or 8 nm wide with various lengths and look like circular “strings of beads” in purified preparations (Figure 2c,e). Whereas all but one [64] non-plant-infecting members of the *Phenuiviridae* have three genomic fragments, most tenuiviruses have four or five fragments (Figure 3). Exceptions include RGSV and the tentative tenuivirus RmSV, both with six fragments, and MeTV, which has eight genomic fragments, the highest number thus far for a tenuivirus. Whereas the genome of tenuiviruses encodes a precursor to glycoproteins, enveloped virus particles have never been observed. For this reason, tenuiviruses are generally known to exist as infectious RNPs. Although one might debate whether or not to regard these as particles, the embedding of all genomic elements within separate infectious RNP units, in contrast to the membrane-bound particles of tospoviruses and emaraviruses, raises the question whether and how to guarantee the fact that neighboring cells are infected with an entire set of all genomic segments. For this very same reason, some even postulate that tenuiviruses present multipartite viruses, i.e., each segment is encapsidated in separate particles, a situation common for plant viruses but very rare in viruses infecting other non-plant systems [65]. The 5′ and 3′ extremities of all genomic RNA segments share 10 complementary terminal nucleotides (ACACAAAGUC), which are conserved among tenuiviruses and partially shared with other phenuivirids, but also an additional 7–15 nucleotides, with a looping out of one C residue at position 11 in one of the extremities [14]. The RNAs encode either one ORF in negative polarity, or two ORFs in ambisense orientation. The genomic segments are numbered by decreasing size, among which four to five show similarity among tenuiviruses, whereas other segments are unique to an individual virus (Figure 3).

For tenuiviruses, like all other members of the *Bunyavirales*, the largest segment (RNA1) of about 9 kb encodes the RdRp (≈340 kDa), required for virus replication (Figure 3). The RdRp also contains endonuclease activity required for cap-snatching [40,41]. The RdRp is associated with RNPs of RSV, as commonly observed for polymerases of NSVs. Both RGSV and EWSMV encode a second small protein pV1 in a virion sense, although there is no obvious similarity between the two proteins of these viruses (nowadays, and as applied in this overview and figures of the genome organizations, tenuivirus-encoded proteins are more generally referred to as pC and p (or pV), and V and C referring to their encoding by the viral (v) or viral complementary (c) genomic strands. In older literature, many of these (non-structural (NS)) protein genes were often referred to as NS (v-strand encoded) or NSvc (vc-strand encoded)). The pV1 protein of EWSMV, but not of RGSV, presents a domain similar to plant NAC-like transcription factors that regulates genes involved in plant development and in responses to biotic and abiotic stresses [17].

Except MeTV and RmSV, all tenuiviruses contain a RNA2 segment of ≈3.5–4 kb, encoding two proteins in ambisense orientation, pC2 and p2, that also appear to be homologous between tenuiviruses. The pC2 protein (also referred to as NSvc2 in many papers) is an insoluble protein that matures from ER to the Golgi complex via COPII-dependent vesicle transport [66] and acts as a helper component in insect transmission [67]. The protein shows some similarity to the GP encoded by the M RNA of uukuviruses (*Phenuiviridae*). The p2 encoded on the viral genomic strand is a weak suppressor of RNA silencing in plants and is thought to promote systemic infection through interaction with nucleolar fibrillarin.

RNA3 of most tenuiviruses and the functional/genetical homologous RNA5 of RGSV (≈2–2.7 kb) encodes two proteins in ambisense orientation (Figure 3). The one encoded by the viral genomic strand is p3 or NS3 (non-structural protein 3) and functions as a silencing suppressor (see below), and the protein encoded by the viral complementary strand is the structural protein pC3 (N or NC). For RGSV RNA5, the functional homologous genes are denoted p5 and pC5 (or N), respectively (Figure 3).

RSV RNA4 and the functional homologous RNA6 of RGSV (≈1.9 to 2.5 kb) encode two proteins: the major non-capsid protein p4 (or NS4) that accumulates in infected cells, forming amorphous inclusions and needle-like structures typical of tenuivirus infection, and pC4 or NSvc4/MP involved in virus movement in infected plants. For RGSV, these are, likewise, referred to as p6 and pC6 (MP), respectively (Figure 3).

RNA 5 (≈1300 nt) present in MSpV and EHBV encodes a highly basic hydrophilic protein (≈44 kDa) of unknown function.

RNAs 3 and 4 of RGSV (3.1 and 2.9 kb, respectively) each code for two proteins in ambisense orientation, but both proteins show no similarity to other tenuivirus proteins and their functions remain unknown (Figure 3).

The dicot infecting MeTV and the tentative tenuivirus RmSV differ from the other monocot-infecting tenuiviruses in their genome organization (Figure 3). Besides the 9-kb RNA1, encoding the RdRp, they possess seven and five additional RNA segments, respectively, of 1.2 to 1.8 kb that either encode one or, in ambisense gene arrangement, two proteins. The proteins encoded by RNA4 of MeTV, and by RNAs 2 and 5 of RmSV are similar to the N (NP) protein of the other tenuiviruses. RNAs 5, 6, and 7 of MeTV show some similarity to RNAs 3, 4, and 6 of RmSV, but they present no similarity to any other tenuivirus proteins. The function of all these proteins remains unknown.

The complementary extremities of each tenuiviral RNA segment, similar as described for orthotopoviruses and common for all segmented NSVs, enable base pairing and thereby forming a stable panhandle structure, which explains the circular appearance of their RNPs (Figure 2c) [1]. These panhandle structures are important for replication and transcription, as in animal-infecting members of the *Phenuiviridae* [68]. All components required and being part of the replicase complex of tenuiviruses are not well known. The host chaperone HSP70 was shown to be necessary for RSV infection and to interact with the N-terminus of RSV RdRp, indicating that HSP70 probably plays a role in viral replication [69]. Tenuiviruses replicate both in plants and insects, but their replication and gene expression patterns display a clear difference related to host specificity. In plants, the ratios of all four RSV segments varied by no more than 15-fold, but in planthoppers, a 300-fold difference was observed between segments [70]. Along the same line, the expression patterns of the seven genes were also different between plants and insects [70]. Earlier studies had already shown the effects of panhandle structure (de)stabilization on replication/transcription of the animal-infecting phenuivirid *Uukuniemi* (UUK) [71], but also on the possibility to repair genomic termini [72]. During replication of genomic RNA segments in plants and insects, extensions of 16 and 15 nt, respectively, are found at the 3′ extremity of RSV segments 1 and 2, which seem to become progressively eliminated after switching to plant hosts [73]. If and how the extensions on RSV RNA segments would affect their replication by compromising the formation of the panhandle structure and its replication/transcription promoter activity is not known [74]. A recent study showed the targeting of these extensions by an endogenous insect microRNA (miR-263a) and repressing the inhibitory effect of the extensions on viral promoter activity in insects [75].

As with other viruses from the bunyavirales, tenuivirus proteins are expressed from subgenomic or near-genomic length viral mRNAs that are transcriptionally initiated by cap-snatching, similar to tospoviruses (for a review on cap-snatching, see [76]). To this end, the N-terminal part of the RdRp contains endonuclease activity [41] consistent with the RdRps from other viruses that employ cap-snatching. As a consequence, the 5′ extremities of tenuivirus mRNAs are heterogeneous and differ from those of the genomic RNAs [77]. How a snatched capped RNA leader primes transcription initiation is not clear. The “base-pairing” model indicates that the 3′ terminal residues of the capped RNA leader base pair with at least one of the first nucleotide of the viral template. The “prime-and-realign” model proposes that capped RNA leaders, after being extended for one or a few nucleotides, shift backwards to realign on the first 3′ terminal residues of the viral template and re-elongate. For RSV, the latter has been observed quite frequently and more than with RGSV, especially with short-capped RNA leaders. It is therefore postulated to convert short leaders into more suitably sized primers for elongation [77,78]. High-throughput sequencing analysis of non-viral leader sequences from tenuivirus mRNAs also showed frequent targeting of host cellular mRNAs encoding translation- and photosynthesis-related proteins [77]. Besides endonuclease and polymerase activity within tenuivirus RdRps, the RSV RdRp furthermore contains deubiquitination activity [79]. The biological significance of this activity for the virus infection cycle is unknown, although a recent study has shown that the accumulation of RSV in the planthopper is inhibited by ubiquitin-conjugating enzyme E2 [80].

How plant viruses induce symptoms in their hosts has not been completely elucidated. Symptoms appear as consequence of infection due to the hijacking of important plant functions by viral components. For tenuiviruses, RGSV p5 protein is shown to interfere with the CBL–CIPK Ca^2+^ signaling network involved in the regulation of ion homeostasis. RGSV-infected plants show a significant decrease of potassium content, whereas some RGSV symptoms mimic potassium deficiency [81]. It has also been reported that virus infection interferes with plant hormone homeostasis and affects plant development. RSV p2 directly interacts with rice auxin response transcription factor OsARF17, a modulator of auxin signaling, and interferes with its DNA binding activity, making the plants more susceptible to viruses [82]. RSV infection hijacks brassinosteroid signaling pathway in rice, suppressing jasmonic acid-mediated resistance [83]. Upon RGSV infection, genes associated with tillering and genes involved in the inactivation of gibberellic acid and indole-3-acetic acid were activated, which may account for the excess of tillering and the stunting observed during RGSV infection [84]. The RGSV p3 has been proven to induce a E3 ubiquitin ligase (named P3-inducible protein 1 (P3IP1)) that triggers ubiquitin–proteasome-mediated degradation of the NRPD1 subunit of plant-specific RNA polymerase IV, which is necessary for RNA-directed DNA methylation. Transgenic expression of RGSV p3 or knockdown of NRDP1 resulted in abnormal development similar to RGSV symptoms in rice [85]. A region of RSV RNA4 was observed to be (partially) complementary to a sequence of the eukaryotic translation initiation factor eIF4A gene. Virus-derived small interfering (vsiRNAs) from that region are found in infected *N. benthamiana* that potentially could target eIF4A mRNA for regulation, causing leaf twisting and stunting [86].

Chloroplasts play an important role in virus infection, and virus-induced alteration of chloroplasts is often associated with photosynthesis defects and chlorotic symptoms [86,87]. Genes related to chlorophyll synthesis are predominantly suppressed by RGSV infection [84]. RSV infection also disturbs chloroplast targeting of host proteins [88,89,90], and the downregulation of chloroplast genes by RSV infection has been shown to be associated with chlorosis [86].

### 2.3. Emaraviruses

Emaravirus particles resemble those of orthotospoviruses, although they appear distinct on one point. In most emaraviruses, double-membrane-bound bodies (DMBs)/particles, ranging from 80 to 200 nm in diameter, have been observed (Figure 2d). Within the infected cell, they often localize near the ER and Golgi. Moreover, flexuous structures, 3–10 nm in diameter, and resembling the RNPs of orthotospoviruses and tenuiviruses, have been collected from infected tissues (Figure 2e) [19].

Within the figure on genome organizations, the emaravirus HPWMV genome is taken as a reference, with eight genome segments (Figure 3), each encoding one ORF in negative polarity; however, across all described species, the genome segments vary in number between 5 to 10 segments. For this reason, one might debate on what is a reference genome for emaraviruses, and in the future a situation establishes with similarity to tenuiviruses (Figure 3). For example, *Actinidia chlorotic ringspot-associated virus* (AcCRAV) and *Pigeon pea sterility mosaic virus* (PPSMV-1) have only five RNA segments [91,92]. Across all species, the RNAs 1 through 4 are highly conserved and are numbered by decreasing size, while the segments RNA 5 through 10 vary between 1000 and 1700 nts, are not ordered by size, appear genome segment variants or duplications, and their occurrence varies among emaravirus species (Figure 3). This makes the counting and numbering of a reference set of genome segments a difficult and challenging issue with emaraviruses, and for which a clear definition may be needed. 

As for tenuiviruses, RNA1 encodes the viral RdRp, RNA2 encodes the envelop GP precursor, RNA3 encodes the viral N protein, and RNA4 encodes the viral MP (Figure 3). The RdRp is usually between 260 and 270 kDa and shares significant homology with the counterparts of bunyaviruses. The N-terminal region contains endonuclease domain and is suggested to function similar to the orthotospovirus endonuclease in cap-snatching from cellular mRNAs [40]. Evidence for cap-snatching was provided in studies of fig mosaic virus (FMV). Polyribosomal RNA was isolated from infected fig leaves, and 5′ rapid amplification of cDNA ends (RACE) was performed identifying 12–18 nt of non-viral RNA sequences at the 5′ end of FMV RNAs [93]. The polymerase active site consists of six motifs known as the preA, and A through E motifs that lie toward the C-terminal half of the RdRp [23,94,95]. The importance of this region for viral RNA synthesis was confirmed by introducing a mutation into the rose rosette virus (RRV) RdRp that debilitated RNA synthesis [95]. The C-terminal region has a domain that is suggested to bind 5′ 7-methylguanosine cap structures, although this has not been shown experimentally [23,96].

Envelope glycoproteins Gn and Gc are derived from the GP precursor encoded by RNA2 (Figure 3). Among emaraviruses, the mature envelope glycoproteins appear to have three transmembrane domains, but the numbers of glycosylation sites vary. Unlike orthotospoviruses that have two mature envelope glycoproteins, emaraviruses may have two or three mature glycoproteins. PPSMV-1 has a single peptide cleavage site, FS_201_/D_202_D, predicted to yield two mature glycoproteins Gn (22.4 kDa) and Gc (51.6 kDa) [96]. Analysis of the *Juju yellow mosaic virus* GP suggests that there are two protease cleavage sites, V_25_ES/SS and V_218_LA/DD. Processing of the precursor polyprotein produces three mature glycoproteins, Gn (22.3 kDa), Gs (3.0 kDa), and Gc (50.89 kDa) [97]. For AcCrAV, the two predicted cleavage sites are VNT_23_/K_24_V and VKA_196_/E_197_D and are predicted to produce Gn (19.8 kDa), Gs (2.6 kDa), and Gc (53.1 kDa) [92]. Whether Gs shows functional similarity to the animal-infecting bunyavirus GP-derived NSm protein [98] is not known.

RNA3 encodes the N protein of 32–37 kDa, and can vary from 12 to 80% amino acid sequence identity between species (Figure 3). At the amino acid level, there are three conserved blocks of amino acids, namely, NV(L/V)S(F/Y)NK, NRLA, and GYEF, predicted to be involved in RNA binding [99,100,101]. Cellular studies of the FMV nucleocapsid showed localization and motility along the endoplasmic reticulum (ER). Electron mobility shift assays demonstrated N protein-binding viral RNAs. The N coats the viral RNA, which is then surrounded by double membrane envelope that protrudes through the ER. Nucleocapsids form aggregates in cells, and their motility depends upon the actinomyosin system [102]. Interestingly three species seem to have two versions of RNA3—HPWMoV, Perilla mosaic virus (PerMV), and Pistacia mosaic virus (PiMV)—the latter of which stands out as a recently identified species that is described as having two distinct versions of RNA3, which for PerMV are named RNA3a and RNA3b [103,104,105].

RNA4 encodes the MP of approximately 27 kDa. Studies using Raspberry leaf blotch virus (RLBV) and FMV showed that each P4 protein could rescue a movement-defective potato virus X that lacks a portion of the TGB1 movement protein [106,107,108]. Across emaravirus species, the P5 through P10 proteins show low sequence similarity, and the functionally identified proteins may not be conserved across species. In addition, the occurrence and function of additional RNA5 and RNA6 vary among different emaravirus species. Two representatives of contrasts among species are RRV and HPWMV whose genomes contain seven and eight genome segments, respectively. Another unique feature of emaravirus genomes is that the RNA 5 through 10 segments appear redundant in terms of encoding highly similar proteins. One example is PerMV, which has three versions of RNA6 named RNA6a, RNA6b, and RNA6c [104]. PiMV has two variants of RNA5 named RNA5a and RNA5b. For PiMV and RRV, the RNA5 and RNA7 share between 37% and 40% identity and the P5 and P7 proteins share approximately 40 to 73% identity [103,109].

The 3′ and 5′ termini of all RNA segments are highly conserved and show inverted complementarity, thereby enabling the formation of a panhandle (Figure 2e) and containing promoter activity for replication and transcription. The consensus sequence GGAGAACACUACU at the 3′ terminus and the AGUAGUGAACUCC at the 5′ terminus of each genome segment is conserved across all members of the *Fimoviridae* and share identity with the animal- and insect-infecting *Peribunyaviridae* and *Cruliviridae* and two genera of *Phasmaviridae* (*Feravirus* and *Jonvirus*) [23]. The endonuclease domain in the viral RdRp, along with the potential panhandle structure of the terminal genome sequences, suggests a cap-snatching model for RNA synthesis. However, unlike orthotospoviruses and tenuiviruses, extraction of stable high-molecular-weight dsRNAs have been reported for RLBV, RRV, FMV, PPSMV-1, PPSMV-2, TiRsAV, Alfalfa ringspot-associated virus (AraV), Aspen mosaic-associated virus (AsMaV), and others [25,95,96,107,110,111,112]. In fact, dsRNA isolation technology is becoming commonplace for HTS approaches to identify new tentative emaravirus species. This is unexpected for NSV, for which the general overarching model of replication is that the viral genomic (or v) and antigenomic (or vc) RNAs are never naked but always encapsidated by the N protein. The only model to explain the accumulation of dsRNAs applies to positive-strand RNA viruses for which the 3′ end of the genomic RNA creates a transient RNA primer for reverse transcription and extension of the anti-genomic RNA producing the double-stranded (ds) replicative form. This model for negative strand RNA virus genome synthesis might not apply to emaraviruses, for which there are several reports of dsRNA accumulating. Further research is needed to learn if the replicative strategy for emaraviruses occurs *de novo* or involves endogenous priming.

## 3. The Role of Non-Structural, Accessory Proteins in Host Adaptation: Inter/Intracellular Movement and Counter-Defense of Antiviral RNAi

### 3.1. Orthotospoviruses

Orthotospoviruses show similarities in genome organization, encoding proteins (RdRp, N, GP) and their functions, and expression strategy to homologs from the animal-infecting members of the *Bunyavirales*. However, they encode one additional non-structural accessory protein (NSm, in which m refers to its encoding by the M RNA segment (Figure 3)), in a completely separate ORF, absent in animal-infecting counterparts. Although a similarly named protein is found in the latter group and is processed from the GP precursor, it has a completely different function. The orthotospovirus NSm protein is involved in the adaptation of orthotospoviruses to infect plant hosts, i.e., it presents the viral MP, a requirement that applies to all systemic plant viruses to allow cell-to-cell movement of infectious viral entity, mostly through modification of plasmodesmata, the channels that connect the cytoplasm of two neighboring cells [113]. Orthotospovirus NSm enables the intercellular movement of infectious RNPs via a tubule-guided manner through plasmodesmata [29,114]. NSm-mediated RNP transport involves an interaction between NSm and N protein [115,116,117,118,119]. The protein is only transiently expressed during early stages of the infection cycle in planta and during that time not only localizes at plasmodesmata but also associates with cytoplasmic localizing RNPs [29]. Microinjection studies of fluorescing dyes in stable tobacco transformants expressing NSm show diffusion of LYC-dextran (10 kDa) molecules via NSm-modified plasmodesmata, but this is not observed in untransformed tobacco plants, indicating that NSm modifies/enlarges the size exclusion limit of plasmodesmata [120].

During the intra/intercellular trafficking, NSm interacts with ER chaperones from the DnaJ protein family [115], co-chaperone SGT1 [121], and At-4/1 [122]. The latter protein is found in ER-derived membrane structures nearby the plasmodesmata and is able to independently move intra- and intercellularly. NSm physically interacts with ER membranes; however, it does not rely on the ER to Golgi transport pathway, nor the cytoskeleton for its trafficking [123]. Mutations in NSm that impair the interaction with ER inhibit cell-to-cell movement [123]. Furthermore, the C-terminal end of NSm is essential for orthotospovirus movement, as mutants lacking this domain do not interact with N, nor are they able to form tubular structures [118,119].

Although NSm assists in cell-to-cell movement of viral entity in a tubule-guided manner, it is also able to complement movement-deficient Tobacco mosaic virus (TMV) in cell-to-cell and long-distance movement [58,124]. TMV does not rely on a tubule-guided cell-to-cell transport, nor does it move as mature particles, and therefore these data imply that orthotospovirus NSm is able to support long-distance movement of viral RNAs. The C-terminus of NSm appears to be essential for this movement [124]. Similar rescuing of a movement-deficient Cucumber mosaic virus (CMV) or Alfalfa mosaic virus (AlMV) by complementation with TSWV NSm has been observed [119,125].

**Figure 4:**
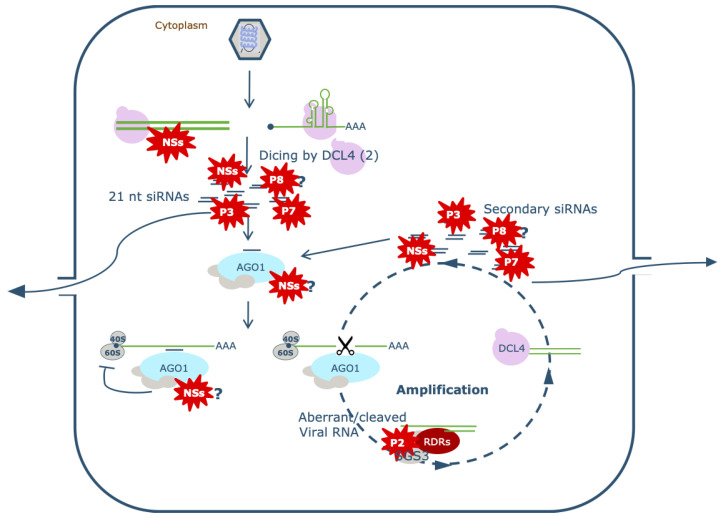
Antiviral RNAi and viral counterdefense used by viral suppressors of RNAi (VSR). Identified and putative VSRs are indicated for tospoviruses (NSs), tenuiviruses (p2 (RGSV, RSV) and p3/NS3 (RHBV, RSV)), and emaraviruses (p7 and p8 (HPWMoV)). Ago, Argonaute; DCL, Dicer-like; RDR, RNA-dependent RNA polymerase; siRNA, small interfering RNA (for an overview on (antiviral) RNAi, see [10]).

The second non-structural protein (NSs, in which s refers to its encoding by the S RNA segment (Figure 3)), like the ortholog from the animal-infecting relatives within the *Peribunyaviridae* and *Phenuiviridae*, is involved in the modulation of host antiviral defense responses. While in animal systems this involves antagonistic properties of interferon-induced defense responses and shut-off of host cell protein synthesis [126], in plants, the orthotospovirus NSs protein acts as a suppressor of the antiviral RNAi defense response [10] (Figure 4). RNA interference (RNAi, and also known as RNA silencing) is a conserved eukaryotic gene regulatory mechanism that also acts as an antiviral defense mechanism. It is triggered by double-stranded (ds)RNA structures that arise during viral infections (viral dsRNA intermediates or intramolecular folding structures) that are recognized and processed by Dicer enzymes (in plants, Dicer-like (DCL)) into viral small interfering RNAs (siRNAs) approximately 21 nt in size. From these, one strand is loaded in an argoaute (AGO) protein, the effector component of the RNA-induced silencing complex (RISC). Using this guide strand, RISC recognizes (viral) RNA target sequences with sequence complementarity and subsequently cleaves/degrades the RNA by AGO slicer activity [10].

The NSs protein is able to suppress local and systemic silencing of GFP [127,128,129,130,131] and exerts RNA silencing suppressor activity by binding long double-stranded (ds)RNA and small interfering (si)RNAs, thereby preventing their processing by Dicer-like proteins (DCLs) into siRNAs and the activation of antiviral RNA-induced silencing complexes (RISCs), respectively [132,133] (Figure 4). 

The protein is also able to bind the structurally similar micro (mi)RNAs and thereby interfere in host gene regulation and plant development [132]. This agrees with observations made in tomato plants infected with Groundnut bud necrosis tospovirus (GBNV), in which the NSs protein affects miR319 regulation of the transcription factor TCP1 and controls leaf senescence [130].

Mutation of a WG/GW motif, known to enable Argonaute (Ago) binding (the core component of RISC), compromises the ability of TSWV NSs to suppress local RNA silencing, but not systemic silencing, and implies that this mutant is still binding siRNAs and thereby prevents systemic silencing [129,134,135]. Interestingly, this motif is not found widely spread among orthotospovirus NSs proteins and raises the question as to whether TSWV NSs indeed compromise antiviral RISC activity via Argonaute binding, as well as whether this is generic to all orthotospoviruses (Figure 4).

Alanine substitution analysis of TSWV NSs showed the importance of the N terminal domain in RNA silencing suppression [134], while mutations in a putative alpha helix (amino acids 338–369) within the C-terminal part of the NSs protein from *Watermelon silver mottle orthotospovirus* (WSMV) indicated the importance of this domain in self-interaction and RNAi suppressor activity [131,136], although these latter mutants still retained the ability to bind siRNAs. The GBNV NSs protein furthermore has been shown to exhibit helicase, ATPase, and 5’ α phosphatase activity, in which the ability to suppress RNAi was independent of helicase and ATPase activity [137,138].

In agreement with its role in virulence, the NSs protein is able to restore the pathogenicity of suppression-deficient heterologous viruses and support systemic movement [131,139,140]. In a similar way, during a mixed infection of two orthotospoviruses, NSs is able to trans-complement and facilitate systemic movement of a co-infecting orthotospovirus [141], sometimes even in an otherwise restrictive host [142].

In plants, NSs is localized in the cytoplasm where, depending on the isolate, it is found in fibrillar structures or paracrystalline arrays [52,143]. Only in the case of *Capsicum chlorosis orthotospovirus* (CaCV) has NSs been reported to also localize in the nucleus [58], but its function there remains unknown.

Orthotospoviruses also replicate during their persistent transmission by thrips. After uptake by L1/L2 larval stages, viral proteins can be detected in the foregut, midgut, ligaments, and salivary gland tissues. Whereas in larvae the primary site of replication involves the midgut epithelium (and nearby muscle cells) and tubular salivary glands, this shifts towards the primary salivary glands in adults, and suggests tissue tropism changes with insect development. Within the salivary glands, accumulating amounts of N and NSs proteins are detected, and mature particles can be discerned secreted into the salivary gland ducts [144,145,146,147,148,149]. The non-structural (cell-to-cell movement) NSm is also expressed in thrips tissues but is thought to not have a function during the infection cycle in thrips. The protein localizes in small electron dense (protein) bodies in various tissues but does not from tubular structures as observed in plant cells, despite its ability to induce tubular structures in *Spodoptera frugiperda* insect cells [114]. Furthermore, in thrips, NSm does not associate with viral RNPs nor with gap junctions, the functional equivalence of plasmodesmata [150].

### 3.2. Tenuiviruses

Tenuiviruses encode several non-structural proteins with known or unknown function (Figure 3), of which one is involved in cell-to-cell movement. In RSV, protein pc4 encoded by RNA4 presents the MP (Figure 3). It accumulates close to the cell walls of infected rice leaves and allows intercellular trafficking of the virus [1,151]. RSV pc4 interacts with several host proteins, particularly the chaperon-like HSP20 [1]. It can also bind single-stranded RNA, most likely required to enable viral movement [152]. pc4 interferes with the S-acylation of the remorin (REM1) and induces its autophagic degradation [153]. Remorins are plant-specific membrane-associated proteins involved in cell-to-cell signaling and restriction of virus movement [154,155], of which group 1 remorins are extensively involved in restricting virus trafficking through plasmodesmata [156]. Their overexpression impairs cell-to-cell movement of various RNA viruses, including RSV in rice [153]. A transmembrane domain of pc4 is essential for its localization to plasmodesmata and for its ability to recover the movement of movement-deficient potato virus X [89,152]. RGSV pc6 shows molecular similarity to RSV pc4 and also acts as a MP [157]. RSV pc4 and RGSV pc6 are both targeted to plasmodesmata via the endoplasmic reticulum-to-Golgi secretory system and actin filaments, and VIII-1 myosin is involved in their plasmodesmata targeting [158,159].

Besides cell-to-cell movement, the systemic infection of plants requires that viruses enter the sieve elements and spread through the phloem to infect new organs. Silencing of RSV p2 protein abolished systemic movement in *N. benthamiana*, showing that this protein contributes to long-distance virus spread [160]. Upon transient expression in *N. benthamiana* epidermal cells, P2 (fused with YFP) initially moves to the nucleolus, where it co-localizes with Cajal bodies [161]. At later stages, P2 leaves the nucleolus and forms numerous distinct bright spots in the cytoplasm [160]. P2 interacts with fibrillarin in the nucleolus of infected *N. benthamiana* cells. Fibrillarin depletion affects the nucleolar and cytoplasmic localization of p2-YFP fluorescence and abolishes systemic movement of RSV but not of several positive-stranded viruses [160]. The mechanisms by which p2 may recruit or manipulate nucleolar functions to promote virus systemic infection, however, are not well known yet.

Like nearly all plant viruses, tenuiviruses also encode proteins counteracting plant defenses, particularly anti-viral RNA silencing. The first tenuivirus RNA silencing suppressor identified is the p3 (NS3) protein from RHBV (NS5 for RGSV) [162,163] (Figure 4). RHBV p3 is able to inhibit silencing not only in plant and insect, but also in mammalian cells, where it has even been shown to act cross-kingdom and rescue a Tat-negative HIV mutant [164]. The RSV p3 suppressor of RNAi self-interacts through several motifs, notably at the N-terminus [165]. Disrupting the interaction by mutagenesis abolishes its silencing suppression activity, showing its functional importance [165]. The RHBV p3 protein efficiently binds both siRNAs and miRNAs, with an affinity depending on the size of the target: 21-nt RNAs are bound >100 times more efficiently than 26-nt RNAs [162,166]. On the other hand, the RSV p3 appears to bind dsRNA in a size-independent manner, although small dsRNAs are preferentially bound [167]. By sequestering siRNAs, p3 prevents their uploading and subsequent activation of antiviral RISC, while sequestering of miRNAs leads to interference of host gene regulation. Mutants that lost siRNA binding activity are compromised in their ability to suppress RNAi [166,167]. The protein also modifies the expression of plant endogenous genes [168] via interaction with OsDRB1, an indispensable component of the rice miRNA-processing complex. It acts as a scaffold between OsDRB1 and pri-miRNAs to regulate their association and aid to in vivo processing of pri-miRNAs. In *A. thaliana*, the protein can partially substitute for the function of dsRNA-binding domain (dsRBD) of AtDRB1/AtHYL1 during miRNA biogenesis. As a result, p3 induces the accumulation of several miRNAs, most of which target pivotal genes associated with development or pathogen resistance. Therefore, p3 is postulated to hijack OsDRB 1, a key component of the processing complex, for the biogenesis of miRNAs, and support virus infection and pathogenesis in rice [169]. While p3 counteracts the antiviral RNAi machinery, it is targeted for degradation by different plant defense mechanisms. The ubiquitin-like protein 5 (UBL5) of rice and *N. benthamiana* interacts with p3 and mediates its degradation by the 26S proteasome, participating in plant defense against infection [170]. P3IP, a previously uncharacterized plant protein, also interacts with p3 and mediates its degradation by autophagy. Transgenic overexpression of P3IP in *N. benthamiana* confers resistance to RSV, confirming the role of autophagy in suppressing RSV infection [171]. Besides p3, p2 (NS2) also exhibits (weak) RNA silencing suppressor activity and exerts this activity through interaction with the plant endogenous suppressor of gene silencing SGS3 [172]. A hypothesis is that p2 promotes the degradation of rice SGS3 via ubiquitination and autophagy, as described for the weak silencing suppressor p4 of the rhabdovirus Rice stripe mosaic virus [173] (Figure 4).

After serial mechanical inoculations of EWSMV on *N. benthamiana*, RNA2 encoding the p2 and pc2 proteins surprisingly became lost but without any obvious effects on symptomatology and mechanical transmission efficiency [17]. The reason for this is unknown, but it indicates that p2 is dispensable for EWSMV infectivity in *N. benthamiana*. However, this observation is in contrast with the finding that p2 is required for long-distance movement of RSV [160]. pC2, required for virus movement in the insect body (see paragraph “transmission”), also appears dispensable for EWSMV infection of plants, at least in *N. benthamiana*.

In RGSV, NS5 (p5) protein, similar to RSV p3, functions as an RNA-silencing suppressor [174] (Figure 4). RGSV p2 also shows (weak) RNA silencing suppressor activity [175]. RGSV p5 interacts with itself and with its cognate p3, for which p5 requires both the N-terminal and C-terminal domains [174]. RGSV p5 also interacts with two kinases in the CBL-CIPK Ca^2+^ signaling network, a plant-specific Ca^2+^ sensor-effector module. However, it is not clear whether this is related to the activity of p5 as a silencing suppressor protein [81]. Contrary to RSV p3, RGSV p5 does not have any distinct binding activity with 21-, 22-, or 24-nucleotide small interfering RNA (siRNA) duplexes [176]. RSV p3 and RGSV p5 also differ in their subcellular localization: whereas GFP-fused RGSV p5 disperses mainly in the plant cytoplasm, RSV p3 localizes in both the nucleus and in the cytoplasm. Altogether, tenuiviral RNAi suppressor proteins do not seem to act similarly, as supported by differences in their subcellular localization [176].

### 3.3. Emaraviruses

Emaraviruses encode the RdRp, N, Gn, and Gc proteins as for other plant- and animal-infecting members of the *Bunyavirales*. However, they encode additional non-structural accessory proteins, and there are only a few reports suggesting functions in cell-to-cell movement or silencing suppression, yet the functions of most proteins are not determined experimentally. Until now, the majority of efforts by researchers have been at the epidemiological level and using next-generation sequencing technology to discover new species members of the genus. The recent development of an infectious clone for RRV creates the opportunity for reverse genetic studies to investigate the functions of various RRV proteins and to carry studies of virus–host interactions at the cellular and molecular levels [95].

Some functional studies have identified RNA4 to encode the MP, and amino acid sequence alignments have allowed the extrapolation to suggest all emaravirus RNA4 segments encode the viral MP (Figure 3). The RLBV and FMV P4 proteins were experimentally shown to function as viral MPs through complementation studies using a movement defective PVX or tobacco rattle virus (TRV). GFP-fused P4 proteins were seen to move between leaf epidermal cells, which are also characteristic of plant viral MPs. The FMV P4 localizes to plasmodesmata and potentially assembles into tubule-like structures similar to the tospovirus Nsm protein. Amino acid sequence analyses indicated that these MPs have conserved structural features including an N-terminal signal peptide sequence followed by predicted β-strands and interspersed α-helices that are similar to plant virus MPs of the 30K superfamily [106,108]. All 30K superfamily members have a conserved aspartic acid (D) residue referred to as the “D motif”. The emaraviruses and orthotospoviruses have a common motif surrounding the D motif: F-X-F-P-X(14)-D-X(52–63)-W, while the tenuiviruses have a submotif F-X-F-P-D [23].

For many other species, RNA5 and RNA6 are suggested to encode virus MPs, but their origins seem to vary among species, and experimental investigations testing their functions are lacking (Figure 3). For example, the P5 and P6 proteins associated with a recently identified isolate of EMARaV obtained from *Sorbus intermedia* encode p42.4 kDa and 27 kDa proteins, respectively, sharing significant homology with the FMV P4 MP [112]. The Eurasian aspen mosaic-associated virus P5 protein shares similarities with the EMARaV P4 and the FMV P6 proteins. On the other hand, researchers hesitate to suggest that the PPSMV-2 P5 and P6 proteins are MPs because they do not show relatedness to P4 [96]. HPWMV RNA6 encodes a p6 protein of 492 amino acids in length while RRV RNA6 is suggested to encode two proteins, p6a and p6b, which are 62 and 233 amino acids in length, respectively [24,109]. As already remarked in Section 3.2, the difficulty and challenging issue of counting and numbering of a reference set of genome segments for emaraviruses is not yet helping to solve some of the above discrepancies and questions, but hopefully will become clarified in the future once these issues have been solved and well defined.

The HPWMV RNA7 and RNA8 encode the p7 and p8 silencing suppressor proteins, respectively [177] (Figure 4). The p7 silencing suppressor binds long dsRNA and protects them from dicing to small RNAs, and p8 protects small dsRNAs. RRV encodes seven RNA segments. The RRV RNA5 and RNA7 segments encode proteins of 467 and 465 amino acids in length, respectively, sharing 54.5% identity, suggesting that they may be orthologs or paralogs [95,178]. The RRV p7 protein is not homologous with HPWMV p7 and thus experimental studies are needed to learn the biochemical functions of the RRV p7 protein.

## 4. Transmission

### 4.1. Orthotospoviruses

The spread of orthotospoviruses relies on a complex of interactions between the virus, thrips vector, and host plant, which is additionally influenced by many (a)biotic factors. In many studies on this topic, and to which most studies described in this section relate to, the major species *F. occidentalis* has been used.

Thrips are only able to transmit the virus when they feed on infected plants primarily during their larval L1 or L2 stages [5,146,179,180,181,182]. Acquisition of the virus likely involves receptor-mediated endocytosis into midgut epithelium cells. The mature envelope glycoproteins Gn and Gc are transmembrane proteins that act within the virion envelop as attachment and fusion proteins, respectively. Evidence for the latter comes from the presence of a highly conserved domain in Gc, shared between orthotospoviruses and the animal-infecting viruses from the former *Bunyaviridae*, e.g., Bunyamwera virus [183], pointing towards a functional homology. This domain likely acts as the fusion domain, as earlier demonstrated with Hantavirus Gc membrane fusion studies [184,185,186,187,188]. Recently, the atomic resolution of the soluble part of the TSWV Gn attachment protein has been determined and Gn shown to dimerize [189]. The atomic resolution details for Gn structure and interacting interfaces suggest that Gn homodimerization is an essential building block within the virion envelope. Considering that analysis was performed in the absence of Gc, further studies are needed to understand the constitution of the holo-spike complex, although other studies have shown Gn-Gc heterodimers form through disulfide bonds on the viral membrane. These combined studies led to a model wherein homodimers and heterodimers play a role in TSWV virion assembly [189].

TSWV replicates in the midgut and then disseminates into the thrips body, where the virus next primarily localizes in the salivary glands as the (second) major site of replication [144,182]. Thrips that have acquired the virus during their larval stages become viruliferous vectors able to transmit the virus during the adult stage, mostly for the rest of their life span. When thrips feed as adults on virus-infected plants, the virus remains restricted to the midgut, and adults do not become viruliferous [190,191], implying that the midgut acts as a barrier to virus escape during certain developmental stages.

The viral Gn and Gc form the holo-spike complex enabling transmission of orthotospoviruses by thrips, and mutants hardly producing virus particles, but also accumulating defective-interfering (L-RNA derived) RNA molecules, are compromised in their transmission efficiency [192,193]. Feeding studies using thrips and a solution containing the soluble form of Gn revealed the ability of thrips to acquire and transmit TSWV was significantly inhibited [194,195]. Similarly, feeding thrips on transgenic plants expressing the soluble form of Gn enormously reduced viral transmission efficiencies by thrips, although this did not protect the plants against TSWV infection [196]. In the *Neohydatothrips variabilis*/Soybean vein necrosis orthotospovirus pathosystem, thrips fed on a combination of soluble peptides containing the “RGD” and the “R_229_” motifs, characteristic of cellular attachment domains and present in Gn of several orthotospoviruses from the American clade, reduced virus transmission by 67% [197].

Although about 15 thrips species have been identified as vector for orthotospoviruses, they exhibit a vector competence towards certain orthotospovirus species only, and not all [6]. While the viral spike complex plays an important role in mediating vector acquisition, it is not yet understood which features determine vector specificity. Recent studies that aim to identify host proteins involving virus acquisition have identified six TSWV interaction proteins (TIPs), using the Gn attachment protein as bait, from first instar larvae (L1) [198]. Among these host proteins, some appear to share homology to proteins that associate with the infection cycle of other vector-borne viruses, but their role in the orthotospovirus transmission cycle by thrips still needs to be determined.

Transmission of orthotospoviruses by thrips is (indirectly) affected by many other factors, often involving altered (preferred) feeding behavior on infected plants (versus healthy plants), and in which the thrips (indirectly) benefits from the virus, e.g., increased life span, fecundity, and offspring [199,200,201,202,203,204]. Concerning the latter, virus infection has been observed to cause transcriptome changes in various life stages of thrips, as well as innate immune responses [205,206,207,208,209,210]. Studies on miRNA profiling have also been performed and may help to further understand gene regulation in thrips during the course of virus transmission [211]. Comparative transcriptome analysis of *F. fusca* and *F. tritici*, a vector and non-vector of TSWV, respectively, has revealed some differences, but the relevance of those towards virus transmission still needs to be further investigated [212]. In some cases, however, no clear effects of viral infection on thrips have been observed [213,214].

Data also show that viral infection changes the plant metabolism and defense responses, turning these plants to be more conducive for thrips feeding/colonization [200,201,203,214]. Upon infection, the viral NSs protein suppresses jasmonic acid (JA) accumulation and moreover reduces genes related to terpenoid synthesis and the content of monoterpene volatiles, causing plant hosts to become more attractive for thrips [215,216]. On tomato plants producing higher amounts of acylsucrose, thrips egg-laying decreases, and virus inoculation is suppressed [217].

Although Gn and Gc are indispensable for acquisition and transmission of orthotospoviruses by thrips, the NSs protein also appears needed for persistent infection and transmission. Studies on a collection of NSs-defective TSWV isolates showed that these could not be transmitted by *F. occidentalis* [218]. While those viruses could still be acquired by thrips and observed to reach the salivary glands, viral titers were significantly reduced and led to a loss of transmission. This was likely due to the absence of a functional NSs protein, as well as in thrips needed to suppress antiviral RNAi.

The presence of thrips resistance in plants indirectly affects the acquisition and inoculation of the virus by thrips as well. While transmission from these plants is basically not affected, thrips show a lower fecundity and lower preference for these plants, and beneficial effects on virus transmission may thus be expected in outstanding crops [219]. Altered feeding and survival rates have also been reported with thrips on peanuts containing resistance traits against TSWV [220,221].

Studies on *Thrips tabaci* have indicated the complexity of virus vector competency, in which the clonal type of *T. tabaci* (population (genetic) structure) and a specific interaction with the (local) virus isolate play a major role [222,223,224,225,226,227]. Moreover, studies with *F. occidentalis* indicate the presence of hereditary traits involved in virus vector competence [228].

### 4.2. Tenuiviruses

Tenuiviruses are transmitted horizontally by delphacid planthoppers in a circulative-propagative manner, i.e., the virus multiplies in the insect vector. They are also vertically transmitted by viruliferous females to their offspring [15]. Immunofluorescence microscopy shows that RGSV and RSV first infect the midgut epithelium of the insect, spread into visceral muscle tissues, disseminate in the hemolymph and other organs, and then move to the salivary glands from which virus transmission to plants can occur [229,230,231]. There are some key differences regarding tissue tropism: RGSV infects the principal and accessory salivary glands of its vector but is not found in neural tissues and ovarioles, whereas RSV is found in both the ovarioles and in the principal salivary glands of its vector but appears absent from the accessory salivary glands [230,231,232,233].

Most bunyavirids are enveloped viruses that enter arthropod cells through interaction between virus surface glycoprotein and host receptors, and in some cases, host receptor is identified as lectin or integrin [229]; on the other hand, tenuiviruses are non-enveloped and do not display glycoproteins in what is thought to be the virion [229]. The structural protein pc3, interacting with viral RNA to constitute the RNP [63], is an important determinant both for tenuivirus horizontal and vertical transmission. RSV N (pc3) interacts with at least five vector proteins [229] including the cuticular protein CPR1 and the lipid transport protein vitellogenin [234]. CPR1 appears to bind RSV in the insect and to stabilize virus concentration in the hemolymph, perhaps protecting the virus or helping its movement to the salivary tissues [234]. Vitellogenin, the precursor of egg yolk in oviparous species, is essential for transovarial transmission of RSV [235]. RSV RNPs binds hemocyte-produced vitellogenin [236] and enables them to invade oocytes through a vitellogenin transportation route [229,237], leading to vertical transmission.

Several viral proteins are involved in viral infection of the insect and horizontal transmission. The glycoprotein pc2 (NSvc2) is required for RSV entrance into the planthopper midgut cells. It acts as a helper component for transmission, the first described for a persistent propagative virus [67]. In infected cells, NSvc2 is processed into two mature proteins: an amino-terminal protein (NSvc2-N) and a carboxyl-terminal protein (NSvc2-C) that both interact with RSV RNPs. NSvc2-N binds to an unknown receptor at the surface of midgut lumen via its N-glycosylation sites. Upon recognition, the midgut cells undergo endocytosis followed by compartmentalization of RSV RNPs/NSvc2-N/NSvc-C complexes into early and late endosomes. Under the acidic condition present inside the late endosomes, NSvc2-C undergoes a conformation change that triggers cell membrane fusion, allowing the release of RSV/NSvc2-N complexes from endosomes into the cytosol [67]. Although typical membrane-bound particles, as with all other members of the Bunyavirales, are not observed with tenuiviruses, NSvc2-N and NSvc2-C clearly act as functional homologs of Gn and Gn, enabling receptor-mediated endocytosis and subsequent release of RNPs from endosomes. Considering Gc-mediated fusion of viral and endosomal membranes generates a pore for RNP release from the endosome into the cytoplasm, it remains intriguing how NSvc2-C fusogenic activity leads to release of tenuivirus RNPs into the cytoplasm, knowing these viruses lack a viral membrane. In agreement with a functional homology of NSvc2 with the tospovirus Gc glycoprotein, similar to that observed with the feeding experiment of TSWV Gn to thrips, the RSV NSvc2-N protein is able to block RSV entry/infection of midgut cells from the small brown planthopper vector [67].

Recently, RSV p3 was found to interact with alpha-tubulin2 of the insect vector, mediating the passage of RSV through the midgut and salivary glands [238]. Moreover, NS4 (p4) appears to be involved in virus movement in its vector [230]. During viral infection, NS4 forms fibrillary cytoplasmic inclusions in various tissues of viruliferous planthoppers. Viral RNPs directly interact with these inclusions, and knock-down of RSV NS4 was found to slow virus spread in the insect body without affecting virus replication in cell cultures [230,233].

As in plants, tenuivirus proteins interact with the host components involved in insect defense mechanisms. The angiotensin-converting enzyme of the small brown planthopper (SBPH) appears to play a role in the immune response against RSV transmission by planthoppers, although the mechanism is not well elucidated [239]. RSV N binds the planthopper G protein pathway suppressor 2, resulting in the activation of the c-Jun N-terminal kinase (JNK) pathway, involved in multiple physiological processes; activation of the JNK pathway leads to increased replication of RSV [240]. RSV p3 interacts with the RPN3 subunit of its planthopper vector 26S proteasome, and repression of RPN3 results in higher virus accumulation and transmission. This suggests that the proteasome plays a role in defense against its vectored plant virus, and that a virus component can subvert this defense through interaction with the 26S proteasome subunit RPN3 [241]. RSV infection also reduces the activity of phenoloxidase in the SBPH by 60%. Phenoloxidase is involved in the melanization pathway, one of the major innate immune responses of insects. RSV p3 binds cleavage sites of prephenoloxidase, preventing phenoloxidase activation by a cascade of clip-domain serine proteases and ensuring viral stability in the hemolymph [242].

RSV reduces the fecundity of its vector by changing the expression of developmental genes in embryos [243]. The presence of RSV leads to changes in vector physiology and behavior: nymph development is accelerated, and adult body weight is increased, which may be related to the increased abundance of yeast-like endosymbionts that provide nutritional benefits and changes in feeding behavior, including the increase of saliva secretion time. These changes could counter the negative effects of the reduced fecundity [244]. RSV infection was also shown to stimulate the expression of an olfactory receptor co-receptor (Orco) in infected SBPH, affecting host seeking behavior of the insects [245] and virus spread as a consequence. Moreover, accumulation of jasmonic acid in RSV-infected plants, activating plant defense against the virus, is attractive for the planthopper vector [246], which can contribute to the horizontal spread of virus. Although only partially elucidated, the changes in vector behavior and plant attractivity mediated by tenuivirus infection show the complexity of virus–host–vector interactions.

### 4.3. Emaraviruses

Unlike orthotospoviruses or tenuiviruses, there is little known about the transmission attributes of emaraviruses. This is because the vast majority of the more than 25 emaravirus species or tentative species have been discovered in the past decade and vectors have been identified for less than half of these species. Emaravirids are transmitted by eriophyoid mites, arthropods that are indiscernible to the naked eye as they average 0.2mm in length, and transmission studies are highly recalcitrant [19].

Eriophyoids are largely monophagous, preferring to feed on one type of plant [247], which may explain the seemingly narrow host range for the majority of emaraviruses. Given the recent discovery of emaravirus species and their vectors, only single eriophyid species are associated with a single virus species. Notably, reports indicate that vectoring mites transmit multiple viruses to the same host species, such as PPSMV-1 and PPSMV-2/*Aceria cajani* [248,249]. The wheat curl mite can simultaneously transmit HPWMV and the tritimovirus wheat streak mosaic virus to wheat [250]. As knowledge expands, we may see more cases of multiple eriophyoid species transmitting the same virus or multiple viruses [251]. It is worth noting that emaravirus-infected plants sustain significantly larger numbers of mites compared to emaravirus-free material [252,253], suggesting that emaravirus infection alters host physiology to encourage vector feeding and improve their fecundity.

The transmission mode of emaraviruses is largely unknown. Some emaraviruses are readily transmissible with short acquisition access periods of ≈15 min whereas others require significantly more time, sometimes several days [248]. Once the mites are viruliferous, the inoculation access period varies from minutes to hours. One example is HPWMV, for which the vector can only acquire the virus in the nymph stages, but not as an adult [254]. Given the microscopic size of mites and the difficulty in identifying their developmental stages, it may be that some transmission studies were performed primarily with nymphs whereas others with adults. Once acquired, the virus is retained between molts and can be transmitted for days and possibly the life of the individual, yet it is not present in the mite eggs [254]. These attributes resemble the attributes of orthotospovirus transmission by thrips, except for the short acquisition access period. *Phytoptus pyri*, a common pest of mountain ash, was tested positive for both the genomic and complimentary strands of EMARaV, a possible indicator of virus replication or part of the narrative that emaraviruses encapsidate both v and vc RNAs in their particle. Notwithstanding, a large amount of virus N protein was found to be present in the mite body, altogether leading to the hypothesis that emaraviruses replicate in their vector [255]. In addition, amplification of emaraviruses in individual mites is only possible in vector species, another indication that emaraviruses replicate in their vectors. Still, such observations need to be validated with controlled experiments that follow the timeline of virus accumulation in the mite.

## 5. Evolution

There are recent excellent articles on the phylogeny of the Bunyavirales [23,256], and for this reason, this section focuses on the evolutionary forces that shape the plant-infecting members of the order.

The last common ancestor of the Bunyavirales is most probably an invertebrate, possibly insect-infecting virus [257,258], with none of the plant-infecting taxa being basal to the order [259]. The plant-infecting taxa emerged at different time points, yet they all code for a 30K-like movement protein, possibly obtained from other viruses or hosts [260]. There are clear distinct emergence timepoints with coguviruses and rubodviruses, two recently proposed genera (Table 1), being ancestral to tenuiviruses, whereas orthospoviruses and emaraviruses are present in a different clade of NSV viruses [259].

There are no studies that address how microevolution affects any of the aforementioned taxa, largely because reverse genetics platforms have only recently become available [75,95,261]. On the other hand, there are investigations on how recombination, reassortment, and genome plasticity affect virus macroevolution. These events provide viruses with major fitness gains—acquisition of additional hosts or vectors, but also allowing viruses to evade genetic resistance when it is employed to prevent disease.

Orthotospoviruses, the better studied group of the cohort, have identical genome organization, composed of three RNAs and five proteins expressed using an ambisense strategy (Figure 3). Thrips vectors can be a driving evolutionary force of orthotospoviruses. They feed on many plants, and viruses replicate in their body, providing fertile ground for recombination and reassortment between strains and species [6]. Butkovic et al. [256] identified several recombination signals across the orthotospovirus genome, and along with Oliver and Whitfield [5] have pointed to the diversity of a genus that warrants reclassification to possibly five new genera. Reassortments have also been identified with clear evolutionary implications as shown for a resistance-breaking TSWV isolate as well as the hybrid of Groundnut ringspot and Tomato chlorotic spot orthotospoviruses that can infect tomato [262,263]. There are other studies that indirectly show the advantages presented by reassortment as viruses could expand their host range or acquire additional vectors [117,142].

Tenuiviruses have obvious genome plasticity, with members having genomes ranging from four to eight segments. Whereas the function of the core viral protein has been determined [63,67,90], the roles of the auxilliary proteins in several members is to be examined. Genome segments may be subject to different selection pressures, as nicely observed with EWSMV RNA2. This segment was lost during viral infection after serial mechanical passaging on *N. benthamiana* [17], suggesting that this segment is likely required for vector transmission and dispensable from the plant host. In the case of the better-studied virus in the group, RSV, population structure analysis point to strong purification selection and evidence of recombination [264].

Emaraviruses are the most diverse of the group of the plant-infecting bunyavirids, given that their genome has between 5 and 10 segments and some proteins have no orthologs in the databases. This diversity has led to deliberation on the exact number of segments carried by each virus, and there are cases where emaravirus genomes have been revisited and expanded [22,109]. Emaraviruses with more than seven segments may encapsidate fragments that are products of duplication. As an example, a wheat isolate of HPWMV from Nebraska (HPWMV-NE) was sequenced using partially purified virions and therefore represents the complete genome of the virus. HPWMV-NE has nine segments with two variants of RNA3 present in the same virus preparation [105]. Stewart et al. [265] investigated the diversity of the virus in different hosts and geographic area and determined that those variants were not an artifact of HPWMoV-NE as they were also present in isolates from other geographic areas and hosts.

On the other hand, there are cases where one of the coding regions of the RNA variants has accumulated enough mutations that the duplicated genes become paralogs. PerMV presents an excellent example for both duplication and diversification. The N proteins encoded by the RNA3 variants are homologs sharing over 80% aa identities. The proteins encoded by the RNA6 variants have diversified to the point where these two share about 65% aa identity, whereas the third is much more diverse (<25% aa identity with the other two), presenting a possible paralog [104]. The duplication and diversification events are common and stable, as judged by the sequence of over 90 RRV genomes, all of which had both RNA5 and 7 that code for putative orthologs or paralogs [21].

In addition to the genome plasticity, there is ample evidence that recombination and reassortment are important in the evolution of emaraviruses. In the case of blackberry leaf mottle-associated virus, there is evidence of both recombination and reassortment within the species [266]. In PPSMV-1 and PPSMV-2, two viruses infecting pigeon pea, the reassortment involves segment exchange between viruses [267], illustrating that emaraviruses can evolve fast and possibly combine the attributes of the parent species, as seen in the example of *Groundnut ringspot* and *Tomato chlorotic spot* orthotospoviruses [263].

## 6. Conclusions and Perspectives

Although orthotospoviruses and tenuiviruses have already been known for many decades, and emaraviruses seemingly emerged only recently, the use of HTS/meta-genomics/transcriptomics to resolve plant viromes is boosting the discovery of new isolates/species belonging to the NSVs with segmented genomes. While tenuiviruses and emaraviruses were initially thought to be limited to monocots and perennial plants, respectively, recent HTS efforts have enabled the discovery of tenuiviruses and tenuivirus-like viruses (phenuivirids) in tulip, melon, a plant parasitic nematode, and a fungus [14,268,269,270,271,272], as well as the first emaravirus in an ornamental plant, chrysanthemum [273]. HTS has also led to the discovery of viruses in citrus, with a phylogenetic relation to the *Phenuiviridae*. Due to their unique features, i.e., these viruses have a bisegmented RNA genome of negative and ambisense polarity; encode for the RdRp, N, and MP (but not glycoproteins); and do not contain a viral membrane envelope, they have been classified into a newly proposed *Coguvirus* genus within the *Phenuiviridae* [259,274]. Although studies on woody plants are often more elaborate, the recent discovery of a coguvirus from *Brassica* [275] could boost research efforts to study on these viruses. Likewise, viruses have recently been discovered in apple and grapevine, with a tripartite genome of negative polarity, encoding the RdRp, MP and N proteins, that have been classified into a tentative new genus, *Rubodvirus* [276]. The (global) impact of all these new viruses is yet to be determined.

Thus far, fundamental research on the viruses described in this review has been hampered by the lack of a reverse genetics system, whereas for many animal-infecting NSVs, these have been available for quite some time. Only recently have the first reverse genetics systems been established for TSWV and RRV. The first one was established for TSWV [261], soon followed by the establishment of one for RRV [95]. For both viruses, particles and a systemic infection could be rescued entirely from cDNA clones. However, in contrast to RRV, rescue of TSWV relies on a codon-optimized RdRp and occurs in the presence of various viral suppressors of RNAi, but in which ectopic expression TSWV NSs seemed to interfere negatively. Very recently, a minireplicon system has been established for the RSV tenuivirus in human cells and in planta [75,277,278]. Moreover, with RSV, mini-replicon reporter gene expression was only achieved with a codon-optimized RdRp and was critically dependent on the presence of a viral suppressor of RNAi, but wherein the RSV p3/NSs drastically reduced reporter gene expression. These reverse genetics and replicon systems are expected to boost fundamental research on these viruses, help in the understanding of their disease cycle, and identify targets for future disease management strategies. Until then, one strategy commonly applied by breeders and growers to combat these viruses is to deploy dominant resistance genes. This strategy is problematic, as the number of resistance genes that are available for commercial resistance breeding to combat these viruses is limited, and resistance breaking virus strains emerge [9,12,279]. Consequently, efforts in the past two decades have been aimed toward engineering transgenic resistance strategies on the basis of the exploitation of RNAi or the overexpression of interfering proteins factors [141,280,281]. Due to societal reluctance, the attention has slowly moved to investigate possibilities on topical application of dsRNA molecules [282,283] or to search for alternative strategies to combat virus transmission, e.g., interfere in the transmission cycle of the virus by the insect vector, as exemplified by the use of a soluble Gn or Gn-derived peptides for TSWV and soybean vein necrosis orthotospovirus to inhibit thrips transmission [196,197]. For the above reasons, virus vectors receive growing attention, in which arthropod-infecting viruses could also become tools to control arthropod-borne plant diseases [271]. One topic that has received only a little consideration is the role of microbiota (such as Wolbachia) in modulating virus infection and/or vector-mediated transmission. Understanding the interactions between viruses and the microbiome of the vectoring insect [284] could potentially create new opportunities to combat insect transmitted viral diseases.

Considering the importance of arthropods for virus transmission, we must expand investigations to discover the virome of arthropod species (thrips, aphids, mites, mosquitoes, etc.) [285,286,287,288]. One of the first papers describing viromes in arthropods was reported by Li et al. [284]. This study demonstrated an enormous viral genetic diversity by high-throughput RNA sequencing of 70 arthropod species. Such analyses of the expansive viromes in arthropods has the potential to strengthen the idea of horizontal virus transfer, a concept based on gene module reshuffling between various viruses in (herbivorous) arthropods that contribute to virus evolution and subsequent host speciation (animal vs. plant) [259,289], also indicating the importance of arthropods in viral evolution. It is clear that with the increasing complexity of host viromes, the issue of virus evolution and their role as drivers of evolution becomes more and more interesting, but the multitude of interactions between viruses and their host and vector (and its microbiome) make many of these questions not only increasingly challenging but also more difficult to tackle.

## Figures and Tables

**Table 1:** Taxonomy and classification of plant-infecting NSVs as of 2018. Only families and genera that contain the plant-infecting viruses are specified. The number of genome segments is indicated in parentheses next to the genus name. The total number of currently established species and known vector species for the plant-infecting members of the *Tospoviridae*, *Phenuiviridae*, and *Fimoviridae*, are indicated in parentheses next to the type species and their mode of transmission, respectively. All NSVs are currently assigned in the phylum Negarnaviricota (for “negative RNA”) and subsequently split into subphyla Haploviricotina (for monopartite viruses) and the Polyploviricotina (for segmented viruses).

Negarnaviricota- Subphylum	Class	Order	Family	Genus Containing Plant-Infecting Members	Type Species	Natural Mode of Transmission ****
**Haploviricotina**	Monjiviricetes	Mononegavirales (11 families, 71 genera, 339 species)	Rhabdoviridae (6/30 genera contain plant-infecting viruses)	Alphanucleorhabdovirus (1)	Potato yellow dwarf virus	Arthropods
				Betanucleorhabdovirus (1)	Sonchus yellow net virus	Arthropods
				Gammanucleorhabdovirus (1)	Maize fine streak virus	Arthropods
				Cytorhabdovirus (1)	Lettuce necrotic yellows virus	Arthropods
				Dichoravirus (2) *	Orchid fleck virus	Arthropods
				Varicosavirus (2) *	Lettuce big-vein associated virus	Plasmodiophorid protists ***
	Milneviricetes	Serpentovirales	Aspiviridae (formerly Ophioviridae)	Ophiovirus (4)	Citrus psorosis virus (CPsV)	Plasmodiophorid protists
**Polyploviricotina**	Ellioviricetes	Bunyavirales (12 families, 45 genera and two unassigned genera)	Phenuiviridae (3/19 genera contain plant-infecting viruses)	Tenuivirus (4-8)	Rice stripe virus (8)	Arthropods (14)
				Coguvirus (2) **	Citrus concave gum-associated virus (2)	ND (Grafting) ****
				Rubodvirus (3) **	Apple rubbery wood virus (2)	ND (Grafting) ****
			Tospoviridae	Orthotospovirus (3)	Tomato spotted wilt virus (26)	Arthropods (15)
			Fimoviridae	Emaravirus (5-10)	European Mountain Ash ringspot associated virus (11)	Mites (6)

* Genera containing bi-segmented rhabdoviruses. The order Mononegavirales being defined for viruses with unsegmented genomes, might lead to their reclassification in the future. ** Coguvirus and Rubodvirus are recently proposed genera with similarities to Phenuiviridae *** Plasmodiophorid protists are soilborne obligate biotrophic pathogens of higher plants. **** ND, not determined (many plant viruses, though, can be transmitted also by mechanical inoculation or grafting).
